# Construction and Analysis of High-Density Linkage Map Using High-Throughput Sequencing Data

**DOI:** 10.1371/journal.pone.0098855

**Published:** 2014-06-06

**Authors:** Dongyuan Liu, Chouxian Ma, Weiguo Hong, Long Huang, Min Liu, Hui Liu, Huaping Zeng, Dejing Deng, Huaigen Xin, Jun Song, Chunhua Xu, Xiaowen Sun, Xilin Hou, Xiaowu Wang, Hongkun Zheng

**Affiliations:** 1 Biomarker Technologies Corporation, Beijing, China; 2 Heilongjiang River Fisheries Research Institute, Chinese Academy of Fishery Sciences, Harbin, China; 3 State Key laboratory of Crop Genetic and Germplasm Enhancement, Key Laboratory of Biology and Germplasm Enhancement of Horticultural Crops in East China, Ministry of Agriculture, Nanjing Agricultural University, Nanjing, China; 4 Institute of Vegetables and Flowers, Chinese Academy of Agricultural Sciences (IVF, CAAS), Beijing, China; University of Illinois at Chicago, United States of America

## Abstract

Linkage maps enable the study of important biological questions. The construction of high-density linkage maps appears more feasible since the advent of next-generation sequencing (NGS), which eases SNP discovery and high-throughput genotyping of large population. However, the marker number explosion and genotyping errors from NGS data challenge the computational efficiency and linkage map quality of linkage study methods. Here we report the HighMap method for constructing high-density linkage maps from NGS data. HighMap employs an iterative ordering and error correction strategy based on a k-nearest neighbor algorithm and a Monte Carlo multipoint maximum likelihood algorithm. Simulation study shows HighMap can create a linkage map with three times as many markers as ordering-only methods while offering more accurate marker orders and stable genetic distances. Using HighMap, we constructed a common carp linkage map with 10,004 markers. The singleton rate was less than one-ninth of that generated by JoinMap4.1. Its total map distance was 5,908 cM, consistent with reports on low-density maps. HighMap is an efficient method for constructing high-density, high-quality linkage maps from high-throughput population NGS data. It will facilitate genome assembling, comparative genomic analysis, and QTL studies. HighMap is available at http://highmap.biomarker.com.cn/.

## Introduction

Linkage maps, especially high-density ones, play an important role in the study of genetics and genomics. Application of high-density linkage maps has greatly facilitated discovery of functional genes [1], genome assembly [2]–[6], and comparative analysis of genome structure [7]–[9]. However, most current maps harbor only about hundreds of markers, largely plagued by marker discovery technologies and genotyping costs. The advent of next-generation sequencing (NGS) makes it possible to rapidly discover huge numbers of markers. The genotyping approaches based on NGS, such as SLAF-seq (specific-locus amplified fragment sequencing) [10], RAD (restriction site associated DNA) genotyping [11], and genotyping-by-sequencing [12] are even capable of discovering and genotyping hundreds of thousands of genetic markers throughout the genome at relatively low cost [13]. These revolutionary advances in genotyping technologies provide exciting opportunities to economically construct increasingly dense maps [10], [14], [15]. However, NGS data still inevitably suffer from genotyping errors [16]–[18], especially when sequencing depths are low [19]–[21] and genotypes are highly heterozygous. The inherent features of NGS data impose two major challenges on the construction of high-density linkage map: First, genotyping errors affect the map quality [22]. Second, the marker density explosion leads to the exponential increase in computational intensity [22].

Great efforts have been made to study algorithms for constructing high-density and high-quality linkage map [22]–[24]. RECORD has been developed to produce accurate marker orders in a relatively short time by employing the total number of observable recombination events between adjacent markers as a target function [24]. SMOOTH has been reported to eliminate genotyping errors from genetic linkage data during the mapping process and improve map quality [22]. However, neither RECORD nor SMOOTH is capable of handling populations with high heterozygous loci. OneMap [25] and FsLinkageMap [26] have been developed to construct linkage maps of high heterozygous species. However, OneMap is computationally intensive and FsLinkageMap is incapable of constructing high-density linkage map. JoinMap4.1 employs a Monte Carlo multipoint maximum likelihood algorithm and greatly expedites computational speed in marker ordering [27]; nonetheless, it still suffers from the limit of the marker number in linkage grouping [28], and serious expansion of map distance. The problems caused by genotyping errors and density explosion still remain great challenges for constructing high-density linkage map efficiently and accurately.

Several practical strategies have been used to tackle the difficulties in constructing high-density linkage map in species such as sunflower [29], mouse [7], porcine [30], Brassica napus [31], maize [32], spotted gar [28] and potato [33]. Sunflower linkage map integrated four individual linkage maps [29] to improve marker densities. The integration strategy is laborious and quality suspicious. The linkage map of mouse and pig were constructed by directly using the physical order of marker in the genomes to circumvent the intensive computation of marker ordering [7], [30]. This strategy only works for the construction of species which have genome reference sequence. A bin strategy has been used to construct the linkage map of potato [33], Brassica napus, maize [32] and spotted gar [28]. A “bin” is a group of markers with a unique segregation pattern and is separated from adjacent bins by a single recombination event. The bin strategy reduces computational costs as well as impacts of genotyping errors, but at the cost of incomplete utilization of genotyping data and recombination information, reducing the application value of high-density linkage map. All the above linkage maps enabled the biology studies in these species, but the methods of map construction still suffered from computational time, map quality and the utilization of genotyping data.

Here, by using an iterative ordering and error correction strategy, we present an efficient method that simplifies and enhances the construction of high-density, high-quality linkage map from high-throughput population NGS data (HighMap). Our studies reveal that HighMap has excellent performance of high-density linkage map construction. HighMap provides an important tool for understanding genetics and genomics.

## Material and Methods

All experimental procedures were conducted in conformity with institutional guidelines for the care and use of laboratory animals in Centre for Applied Aquatic Genomics of the Chinese Academy of Fishery Sciences. The protocol was approved by the Committee on the Ethics of Animal Experiments of the Centre for Applied Aquatic Genomics at Chinese Academy of Fishery Sciences (2011AA1004020012).

### HighMap overview

**Figure 1:**
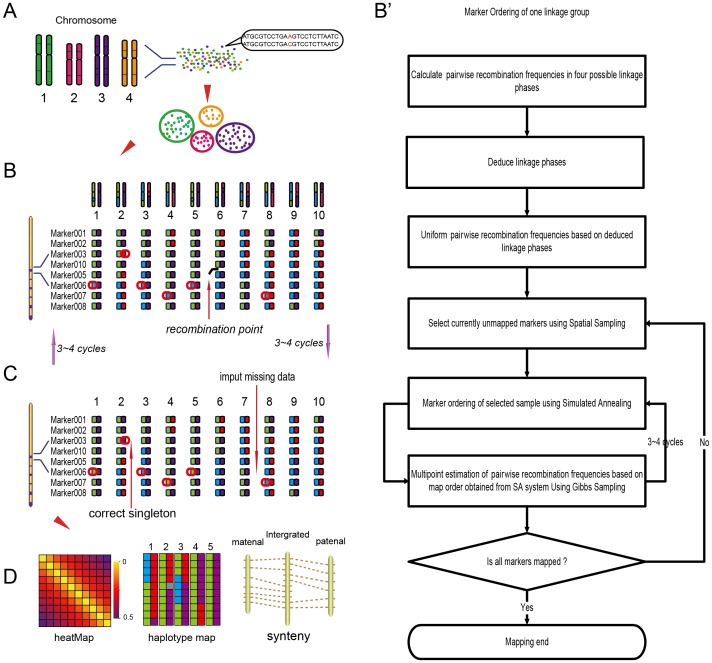
Modules of HighMap algorithm. A: The single-linkage clustering algorithm was used to partition the marker loci into linkage groups based on a pairwise modified independence LOD score for the recombination frequency. B and B': The ordering module combines Gibbs sampling, spatial sampling, and simulated annealing algorithm to order markers and estimate map distances. C: The error correction module identified singletons according to parental contribution of genotypes and eliminated them from the data using *k*-nearest neighbor algorithm. To order markers correctly, the processes of ordering and error correction were carried out iteratively. D: Heat maps and haplotype maps were constructed to evaluate map quality.

Here we report a new strategy, the iterative ordering and error correction, to construct high-density genetic maps. We referred to the error correction strategy of SMOOTH [22], and used a *k*-nearest neighbor algorithm to correct genotyping errors and impute genotyping missing [34]. We employed the enhanced algorithm of Gibbs sampling, spatial sampling and simulated annealing (GSS) [27], [35] to order markers. GSS marker ordering algorithm is computationally efficient [27], but it generates inflated map distances, and has unstable map quality, especially for the data high in genotyping errors. To ensure stability of map quality, we enhanced GSS by using the summation of adjacent recombination fractions (*SARF*) as objective function and adopted Blocked Gibbs sampler after trying different Gibbs sampling methods and different objective functions in simulated annealing. HighMap consists of four modules, designed for linkage grouping, marker ordering, error genotyping correction and map evaluation, respectively (Figure 1). The map evaluation module provides heat mapsand haplotype maps for intuitive displays of map quality [36].

### Linkage grouping

The grouping module uses the single-linkage clustering algorithm to cluster the markers into linkage groups, using a pair-wise modified independence LOD score as distance metric. Assuming a loci pair with segregation type, 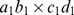 and 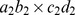, a contingency table of genotypes is produced:
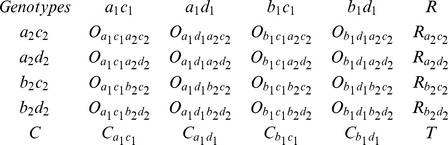



Here 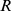, 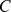, 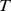, and O represent row-total, column-total, grand-total, and observed number of each genotype, respectively. The expected number 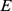 in each cell in above contingency table is calculated by 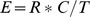. The independence test 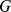 statistic, with degrees of freedom 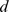 being equal to the number of rows minus 1 multiplied by the number of columns minus 1, is given by 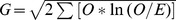. The modified LOD score is obtained from an approximate transformation:
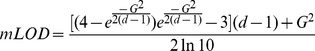



With increasing LOD thresholds, different group nodes form, and these can be represented as a dendrogram, which branches might be huge and deep. Since the algorithm exhaustively searches all possible solutions that the linkage groups number is equal to the chromosome number, it is computationally extensive, especially for marker data of low depth. To speed up the calculation, we simplified the tree structure by adopting two new strategies: deleting small “group fragments”, and merging small descendant nodes into large ancestral nodes.

### Marker ordering and genotyping error correction

The mapping algorithm applies an iterative process of marker ordering and error genotype correction to ensure the accuracy of map order and map distances in the presence of missing observations and genotyping errors (Figure 1). Prior to iterative mapping, recombinant frequencies and LOD scores were calculated by two-point analysis. Based on recombinant fractions and LOD values, linkage phases were inferred by using the anchoring algorithm [37].

Consider a linkage group consisting of 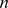 markers, denoted as 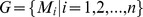. The recombination frequency matrix is as follows: 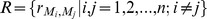. The marker ordering module introduces a combination of statistic techniques, spatial sampling, Gibbs sampling and simulated annealing, to obtain the map order with the summation of recombination frequencies and estimate their mutual genetic distances [27], [35].

In the first stage of the ordering procedure, markers are selected using spatial sampling as following. One marker is taken at random (marker 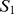) in a priority order of full cross, 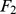 cross and test cross. Markers with a recombination frequency smaller than a given sampling radius 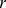 are excluded from the marker set. From the remaining markers, one marker in the abovementioned priority order is selected again (marker 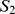). All markers with recombination frequencies smaller than the given value 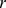 with 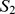 are deleted from the remaining marker set. The sampling process is continued until no markers are left. Finally, a subset of markers 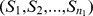 is obtained. In this marker set, all pair-wise recombination frequencies 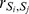 are greater than the given value 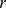.

Subsequently, simulated annealing is employed to find the best map order. We adopted *SARF* as an objective function for it could enormously reduce the computations yet show a lower rate of convergence than the maximum likelihood. Calculation of *SARF* for a given sequence of the above sampled loci is performed by summation of *SARF* in two parent maps, i.e.,
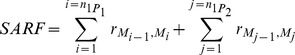



Where 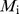 is the 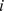 th element of the paternal maps, 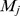 is the 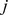 th element of the parental maps, and 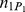 and 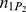 are the number of markers in 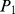 and 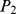 maps respectively. Markers with full cross and 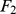 cross segregation pattern occur in both maps, whereas markers with test cross can be observed for that parent only. simulated annealing starts from an initial map order from which the sampled markers are permutated randomly. A new neighbor order is obtained by placing a random locus into a random position, and will be accepted if 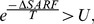
where 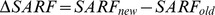, 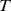 is the acceptance control parameter(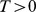) and 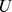 is a random value in the range 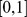. The annealing scheme of 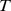 is the same as that illustrated by Jansen and colleagues[35]. The annealing system stops if, in a number of successive steps, the newly generated map order is rejected. Once the optimal map order of the sample markers is obtained, the new order can be used to estimate multipoint recombination frequencies of both parents using Blocked Gibbs sampling, for basic Gibbs sampler often did not work when high-density maps were constructed using small populations or data rich in erroneous markers. The updated recombination frequencies help to integrate the two parental maps, determine the order of test cross markers and optimize the map order in the next cycle of simulated annealing. After three to four cycles, an optimal map of sampled markers is obtained. In the next map-building round, the sampling radius decreases, and a subset of currently unmapped markers is selected and added to the previous sample. The mapping algorithm repeats the previously described stages for the new sample. Then the entire system stops when all markers are in place.

In high-density genetic maps, a genotyping error usually manifests itself as a singleton. A singleton is a single locus in one offspring which is different in parental origin from both its directly neighboring loci [22]. Singletons are mainly caused by erroneous genotypes, but they may also result from other biological phenomena, such as double recombination events, gene conversions, mutations [22]. In the third module, missing genotypes were identified according to parental contribution of genotypes. Identified singletons were eliminated from data and identified missing genotypes were imputed using 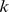-nearest neighbor algorithm [22]. Here 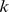 is a parameter that can be specified as appropriate. The number of singletons in each marker reflects marker quality. Markers with singleton ratios exceeding a given threshold are labeled as “suspicious markers”. To avoid that correct markers are deleted by mistake, “suspicious markers” were neither corrected nor imputed. In practice, three to four rounds of ordering and error correction are required to produce a reasonably accurate map order and map distance.

### Simulation study

Simulation data sets were randomly generated based on a full-sib family of an outbreeding species using a Perl script. First, markers were randomly placed along a single paternal or maternal chromosome at random intervals. Then, offspring's genotypes were generated using the simulation data of maternal and paternal chromosomes. Assuming that no crossover interferences occurred, the number of crossover events solely depended on the distance as specified by the simulated positions of the loci on the parent chromosomes. Missing and erroneous data were independently and randomly distributed along chromosomes.

### Experimental data

HighMap performance was further confirmed using the sequencing data of a real full-sib family of common carp which consisted of 211 offsprings [10] (The sequencing data is available at http://highmap.biomarker.com.cn/). JoinMap4.1 was incapable of grouping large data of markers. To compare ordering performance of HighMap with that of JoinMap4.1, we used HighMap rather than JoinMap4.1 to cluster the marker data when we constructed the linkage map using JoinMap4.1.

## Results

### Enhancing utilization of NGS data

Depth and quality of sequencing reads fluctuate randomly across genomes due to sampling randomness. To ensure genotype quality, reads with low depth of sequencing should be discarded in the process of genotype calling [10]. If linkage map software can bear more genotype missing and error, it will be possible to make use of more sequencing data and to create higher-density linkage maps at lower costs. Therefore, an important consideration in this study is NGS data utilization, which reflects the performance of a linkage study method in NGS era. To assess data utilization of HighMap, simulation data were generated from a full-sib family consisting of 200 offsprings. To simulate real NGS data accurately, missing observations and genotyping errors were introduced incrementally and simultaneously as the marker number increased from 100 to 1,000 (Figure S1). The data set was produced by iteratively appending 100 markers, each time with an increment of 5% missing observation and 5% erroneous genotyping.

**Figure 2:**
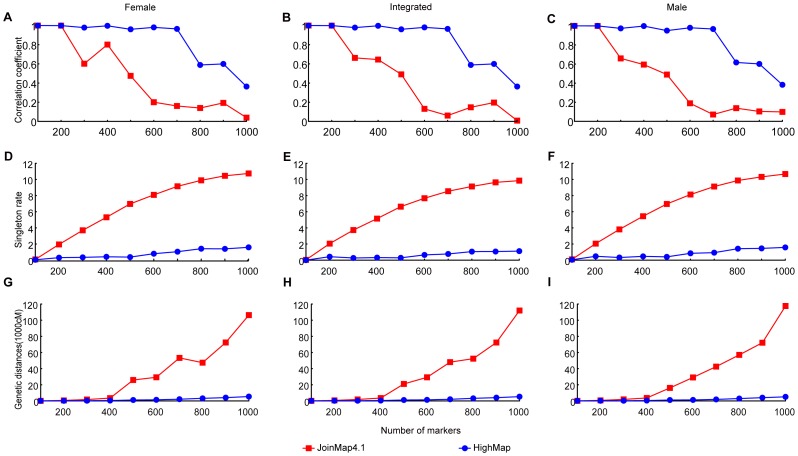
NGS data utilization enhancement by HighMap. The X-axis represents marker numbers. The Y-axis represents Spearman rank correlation coefficient between estimated map marker order and true marker location for A, B and C, singleton rates for D, E and F, estimated genetic map distances for G, H and I, respectively.

Comparative analysis revealed that HighMap permitted the utilization of more markers than JoinMap4.1 (Figure 2A, 2B and 2C). HighMap could make use of 700 markers and create linkage maps with a Spearman rank order correlation coefficient greater than 0.9. In contrast, 300 markers led to the correlation coefficient smaller than 0.8 when the linkage map was constructed using JoinMap4.1. Based on a cutoff value of 0.8 [24], we estimated that HighMap could construct a linkage map with three times the number of markers as JoinMap4.1 could.

HighMap contains an error correction algorithm, which can impute missing observations and eliminate erroneous genotyping from mapping data in the mapping process. Data showed that the algorithm is efficient. Take the dataset of 700 markers as an example. We introduced 10.25% of erroneous data and 12.86% of missing observations (Table 1). After error correction treatment, final erroneous and missing rate reduced to 5.40% and 0.65%, respectively. 94.88% of genotyping errors were accurately corrected, and 89.13% of missing observations were accurately imputed. The performance of HighMap in genotyping error correction and missing observation imputation accounts for its high data utilization.

**Table 1:** Capability of missing imputation and error correction of HighMap.

# of marker	genotyping error				genotyping missing			
	initial rate (%)	% of detected	accurate rate (%)	remain rate (%)	initial rate (%)	% of detected	accurate rate (%)	remain rate (%)
100	0.00	0.00	0.00	0.00	0.00	0.00	0.00	0.00
200	2.37	75.74	97.63	0.88	2.50	85.10	99.53	0.37
300	4.61	82.88	97.65	1.53	5.00	96.33	98.34	0.18
400	6.69	79.15	96.93	2.39	7.50	96.90	97.39	0.23
500	6.32	81.80	97.52	1.99	7.00	97.47	97.60	0.18
600	8.40	77.98	97.14	3.16	10.00	97.42	94.82	0.26
700	10.25	69.42	94.88	5.40	12.86	94.96	89.13	0.65
800	11.90	65.19	93.40	6.91	15.63	93.46	85.75	1.02
900	13.20	60.10	91.07	9.16	18.33	93.27	79.73	1.23
1000	14.35	56.66	89.90	10.42	21.00	91.88	76.44	1.70

A singleton is a single locus in one offspring which is different in parental origin from both its direct neighboring loci [22]. It comprehensively reflects linkage map quality and is useful for quality evaluation of linkage maps since true order of marker is unavailable for a linkage map obtained from real data. When linkage maps were constructed using 700 marker data, the singleton rate of HighMap, 0.77%, was much smaller than that generated byJoinMap4.1, which was 8.54% (Figure 2D, 2E and 2F). This result demonstrates that the correction procedure of HighMap is effective and efficient, which ensures that HighMap with stands high rate of genotyping errors and make more use of marker data.

### Marker order accuracy and map distance stability

**Figure 3:**
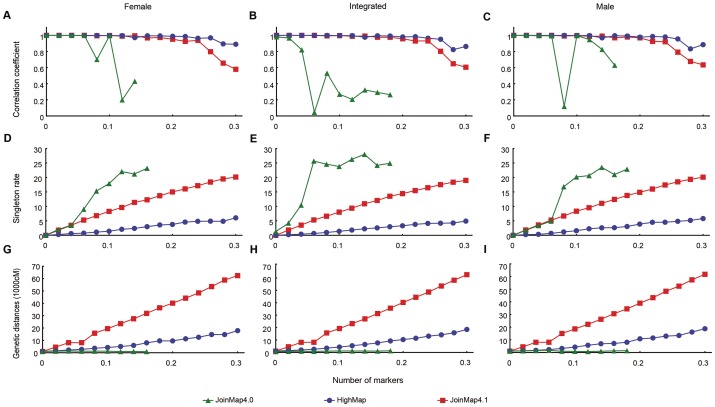
Changes in linkage map quality as genotyping error increased. The X-axis represents genotyping error. The Y-axis represents Spearman rank correlation coefficient between estimated map marker order and true marker location for A, B and C, singleton rates for D, E and F, estimated genetic map distances for G, H and I, respectively.“Integrated”, “Female”, and “Male” indicates integrated, female, or male linkage maps, respectively. JoinMap4.0 failed to construct linkage map due to its inefficiency in estimating linkage phases when the error rate exceeded about 14%.

To assess the performance of HighMap in marker order accuracy and map distance stability, simulation data set was generated from the full-sib family consisting of 200 offsprings with 200 markers, which contained different missing observations or different genotyping errors. Results showed that the Spearman correlation coefficient between the true and calculated marker order based on HighMap decreased less obviously than that of JoinMap4.1 as the marker error rate increased. The differences of the correlation coefficient between HighMap and JoinMap4.1 were more pronounced when error rate exceeded 20% (Figure 3). This result demonstrated that HighMap could offer linkage maps of higher quality than JoinMap4.1 when there were a large proportion of erroneous markers. The singleton rate of HighMap grew slowly as error rates increased, whereas the singleton rate ascended linearly with JoinMap4.1. HighMap led to only 3.3% of the singleton rate when the marker data contained 20% error, whereas JoinMap4.1 led to 14.4% of the singleton rate, suggesting that HighMap detected and eliminated most genotyping errors from the data. Both the correlation and singleton analysis revealed that JoinMap4.0 was sensitive not only to erroneous data but also to missing data (Figure 3 and Figure S2). It failed to construct linkage map due to its inefficiency in estimating linkage phases when the error rate exceeded about 14% (Figure 3). Collectively, HighMap remarkedly outperformed both JoinMap4.0 and JoinMap4.1 with respect to marker order accuracy.

Map distance expansion is mainly caused by genotyping errors and the map distance reflects the quality of a linkage map. In the presence of genotyping errors, it may be necessary to make a balance between controlling the expansion of map distance and ensuring validity of the marker order. We gave the priority to address issue of marker order accuracy for it is more important than the map distance[22]. While ensuring accurate marker order, HighMap greatly curbs map distance expansion. First, map distances that HighMap offered was robust to increasing density of markers. It led to a genetic distance of 2,030 cM when marker numbers reached 700, whereas JoinMap4.1 led to the genetic distance of 48,056 cM (Figure 2G, 2H and 2I). In addition, the map distance estimated by HighMap was insensitive to the increase of genotyping errors. It produced only 430 cM of genetic distance when the data with 10% marker errors was used; whereas JoinMap4.1 gave a genetic distance up to 1,925 cM (Figure 3). These results not only demonstrate that the error correction is an efficient strategy for solving map distance expansion but also account for the reason of better data utilization of HighMap relative to JoinMap.

### Computational efficiency of map construction

Computational efficiency is a concern in linkage mapping. Both grouping and ordering are important in the construction of high-density linkage map. JoinMap still suffers from the limit of marker number in the linkage grouping [28], and this might hamper its application in linkage mapping based on NGS data. HighMap allows to handle hundreds of thousands of markers in relatively short time, for it was developed to construct linkage maps based on NGS data under the Linux operation system, and can also run on a server or cluster. We assessed the efficiency of marker ordering, by comparing it with currently available mapping tools including JionMap4.0 and JionMap4.1, both of which used the default parameters. To this end, we simulated a data set based on a full-sib family consisting of 200 offsprings. The data sets contained markers numbering from 50 to 1,000. The simulation data contained neither missing nor erroneous values. Results showed that computational time consumption increased as marker densities went up (Figure S3). For JoinMap4.0, the time consumption was prohibitively large for the construction of a linkage map with more than 200 markers. HighMap could create a linkage group with 1,000 markers within a single day. We also evaluated the efficiency of marker ordering of OneMap [25] and FsLinkageMap [26]. FsLinkageMap [26] couldn't construct a linkage group with 100 makers; OneMap (using Record, rcd,or ug algorithms) [25] cost more than one day for creating a linkage group with 200 markers (Data not shown). Therefore, FsLinkageMap and OneMap might not be suitable for constructing high-density linkage maps based on NGS data. All experiments were completed on a computer with a Xeon processor (2.4 GHz and 16 Gb memory).

### Application in real population NGS data from common carp

To test HighMap performances on real data, we generated a high-density linkage map of common carp based on a full-sib family NGS data. The integrated map was comprised of 10,004 markers with an average of 11 fold sequencing depths. Among them, 19% were the data with sequencing depths of less than 5 fold. The segregation patterns for these markers are shown in Table S1. Similar to the simulation results, HighMap offered a linkage map of higher quality than JoinMap4.1. Singleton rate of the maps created by HighMap was less than one-ninth of those of the maps constructed by JoinMap4.1 (Table S2). Heat maps (Figure S4) and haplotype maps (Figure S5) verified the quality of the linkage maps that HighMap produced. The linkage maps created by HighMap spanned 5,908 cM in 50 linkage groups, closer to that reported previously [38], smaller than that JoinMap4.1 gave, which was 55,550 cM (Table S3). We also analyzed the Spearman correlation coefficient between the marker order of common carp and the genome sequences of zebrafish [6], a close relative of the common carp. The data revealed that, for about 70% of linkage groups of common carp, the correlation coefficient based on HighMap was larger than those based on JoinMap4.1 (Figure S6), suggesting that HighMap is better than JoinMap4.1with respect to map accuracy.

## Discussion

In this study, we intended to develop a method that can efficiently utilize NGS data and ease the construction of high-density and high-quality linkage map. The challenges of such an effort are associated with the marker density explosion and potential genotyping errors, which involve sequencing depth and sequence heterozygosity. The higher the heterozygosity is, the more the genotyping is prone to error. As was shown in the simulation study, the error rate reached up to 34.1% for markers with ab×cd segregation pattern when markers were sequenced at one fold depth, for markers with ef×eg segregation pattern it arrived at 21.3%, and for markers with hk×hk or nn×np or lm×ll segregation pattern, the error rate stood at 17.4% (Table 2). To address the challenges in the construction of linkage map from high-throughput population NGS data, we exploited an iterative ordering and error correction strategy as well as optimized GSS algorithm. Consequently, HighMap was efficient for constructing high-density linkage maps, even using low-depth sequencing data where genotyping errors and missing observations were common. HighMap offers many advantages over JoinMap4.1. First, the marker order and the map distance are relatively accurate for data with large proportion of missing and erroneous markers. Second, It is robust to genotyping errors, allowing for the use of genotyping data with relatively low sequencing depth and therefore makes it possible to construct high-density linkage map at low cost. The above advantages demonstrate that the iterative marker ordering and error correction strategy is effective and efficient. In addition, HighMap provides an intuitive and convenient way to evaluate map quality in forms of heat maps and haplotype maps. It also has the feature of easy use and does not require the specialized skills.

**Table 2:** Genotyping error and missing rates of different segregation patterns in NGS.

sequencing depths	ab×cd		ef×eg		hk×hk/nn×np/lm×ll	
	error rates (%)	missing rates (%)	error rates (%)	missing rates (%)	error rates (%)	missing rates (%)
1	34.1	43.2	24.7	58.5	17.4	44.8
2	31.7	31.2	23.7	47.6	15.6	31.2
3	25.2	17.8	21.2	36.9	13.6	17.8
4	21.3	11.0	17.6	33.6	10.3	9.2
5	17.5	6.8	12.6	29.4	8.1	6.6
6	14.0	3.4	8.9	28.1	6.4	3.7
7	9.9	2.5	8.4	26.9	5.2	2.6
8	7.6	1.4	5.7	25.9	4.2	2.3
9	5.1	1.0	3.3	25.8	2.6	1.3
10	4.3	0.9	3.4	26.0	2.0	0.6

Linkage maps are widely used in marker-assisted selection, quantitative trait loci mapping, and comparative genome analysis. They are also necessary to anchor scaffolds on chromosomes during genome assembly. Due to the limitation of marker density and population size of linkage maps, there left many scaffolds unanchored or unordered in genome assembly recently published. For example, the cacao genome had only 67% and 50% of assembled sequences anchored and ordered, respectively [39]. The apple (88%, 66%) [40] and grape genomes (69%, 61%) [41] exhibited slightly higher utility of scaffolds. Nonetheless, there was still more than 30% of the scaffold that could not be ordered onto chromosomes. Recently, Hyten et al showed that they can orient additional 23 scaffolds (totaling 7.1 Mb) [42], which were previously unordered, into chromosomes by using a higher-density linkage map with larger size of the population, suggesting construction of higher resolution genetic maps is critical for improving genome assembly. In the case of cacao, the assembled genome was 326 Mb, and scaffold N90 was 75.5 kb [39]. To ensure 90% of the sequence assembly ordered, every 75.5 kb sequence requires at least two markers, and the linkage map should offer at least 8,636 markers in total. The linkage map used in the study had only 1,259 markers [39], about 7,000 markers fewer than what was needed. Therefore there remained up to 2,207 scaffolds unordered in the cacao study. By providing high-density, HighMap will be of great benefit to genome assembly and validation of the scaffold placement on the chromosomes.

In summary, we offer a computationally efficient method for linkage mapping using population NGS data. The development of HighMap should propel the application of NGS in linkage mapping. It is a lasting task to make full use of NGS data at lower cost and to construct high-density linkage maps. Great efforts are guaranteed to further improve the potentials of NGS data utilization in the linkage studies.

## Supporting Information

Figure S1 Simulation data sets containing both the missing and erroneous markers. Missing and erroneous rates increased simultaneously as markers increased from 100 to 1,000.
(TIF)

Figure S2 Changes in linkage map quality as missing observation increased. The X-axis indicates missing observation. The Y-axis indicates Spearman rank correlation coefficient between estimated map marker order and true marker location for A, B and C, singleton rates for D, E and F, estimated genetic map distances for G, H and I, respectively. “Integrated”, “Female”, and “Male” indicate integrated, female, or male linkage maps, respectively.
(TIF)

Figure S3 Computational speed of HighMap. Running time was reported as number of 100 seconds. JoinMap4.0 is computationally demanding when marker data contained more than 200 markers.
(TIF)

Figure S4 Heat maps of pair-wise recombination of the common carp. Yellow color represents tight linkage; red represents weak linkage; blue represents no linkage.
(TIF)

Figure S5 Haplotype maps of the family of common carp consisting of 211 offsprings. Each two columns represent the genotype of an individual. Rows correspond to genetic markers. Green and blue boxes indicate one chromatid from parents; gray boxes indicate missing data.
(JPG)

Figure S6 The difference between the correlation coefficient of HighMap and JoinMap4.1. r_HighMap_ indicates the Spearman correlation coefficient between marker order of linkage map estimated by HighMap and genome sequences of zebra fish. r_JoinMap4.1_ indicates the Spearman correlation coefficient between the marker order of linkage map estimated by JoinMap4.1and the genome sequences of zebra fish.
(JPG)

Table S1 Segregation patterns of common carp linkage map.
(DOC)

Table S2 Singleton rate of common carp linkage map estimated by HighMap and JoinMap4.1.
(DOC)

Table S3 Genetic distance of common carp linkage map estimated by HighMap and JoinMap4.1.
(DOC)
