# Supplementary figures and images for: Construction and Analysis of High-Density Linkage Map Using High-Throughput Sequencing Data
Source: PLoS One. 2014 Jun 6;9(6):e98855. doi: 10.1371/journal.pone.0098855 (PMC4048240; doi:10.1371/journal.pone.0098855)

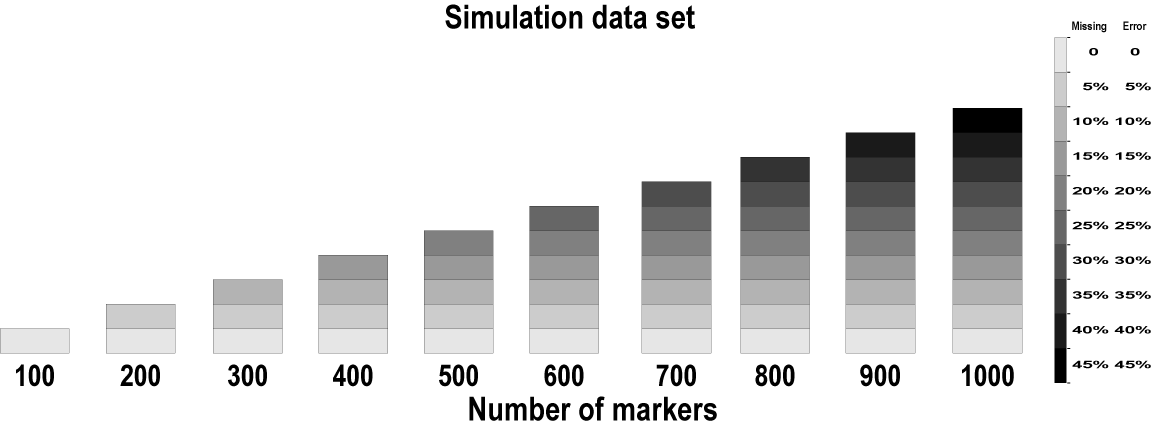

Supplement: Figure S1 — Simulation data sets containing both the missing and erroneous markers. Missing and erroneous rates increased simultaneously as markers increased from 100 to 1,000. (TIF) [file pone.0098855.s001.tif]

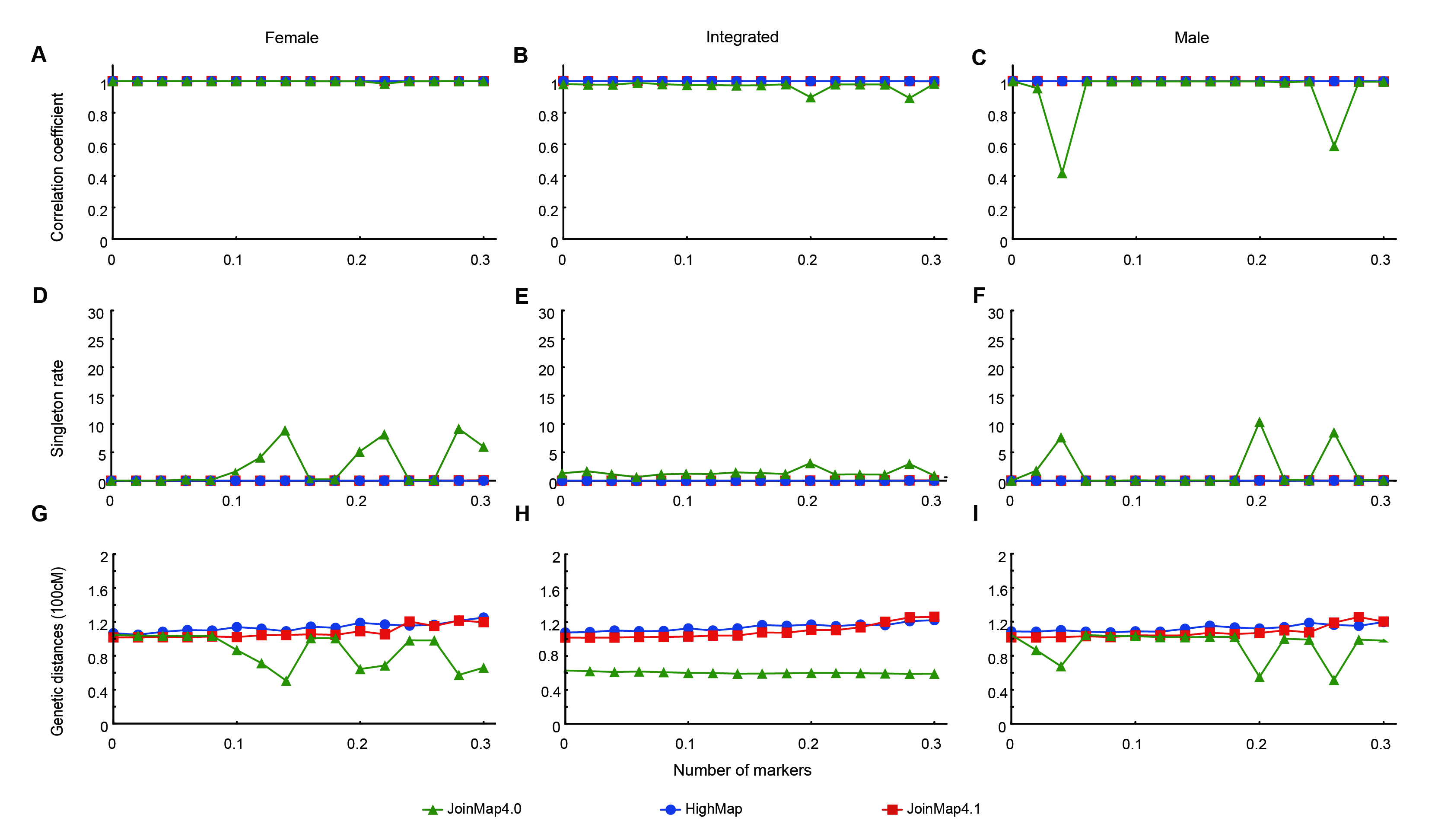

Supplement: Figure S2 — Changes in linkage map quality as missing observation increased. The X-axis indicates missing observation. The Y-axis indicates Spearman rank correlation coefficient between estimated map marker order and true marker location for A, B and C, singleton rates for D, E and F, estimated genetic map distances for G, H and I, respectively. “Integrated”, “Female”, and “Male” indicate integrated, female, or male linkage maps, respectively. (TIF) [file pone.0098855.s002.tif]

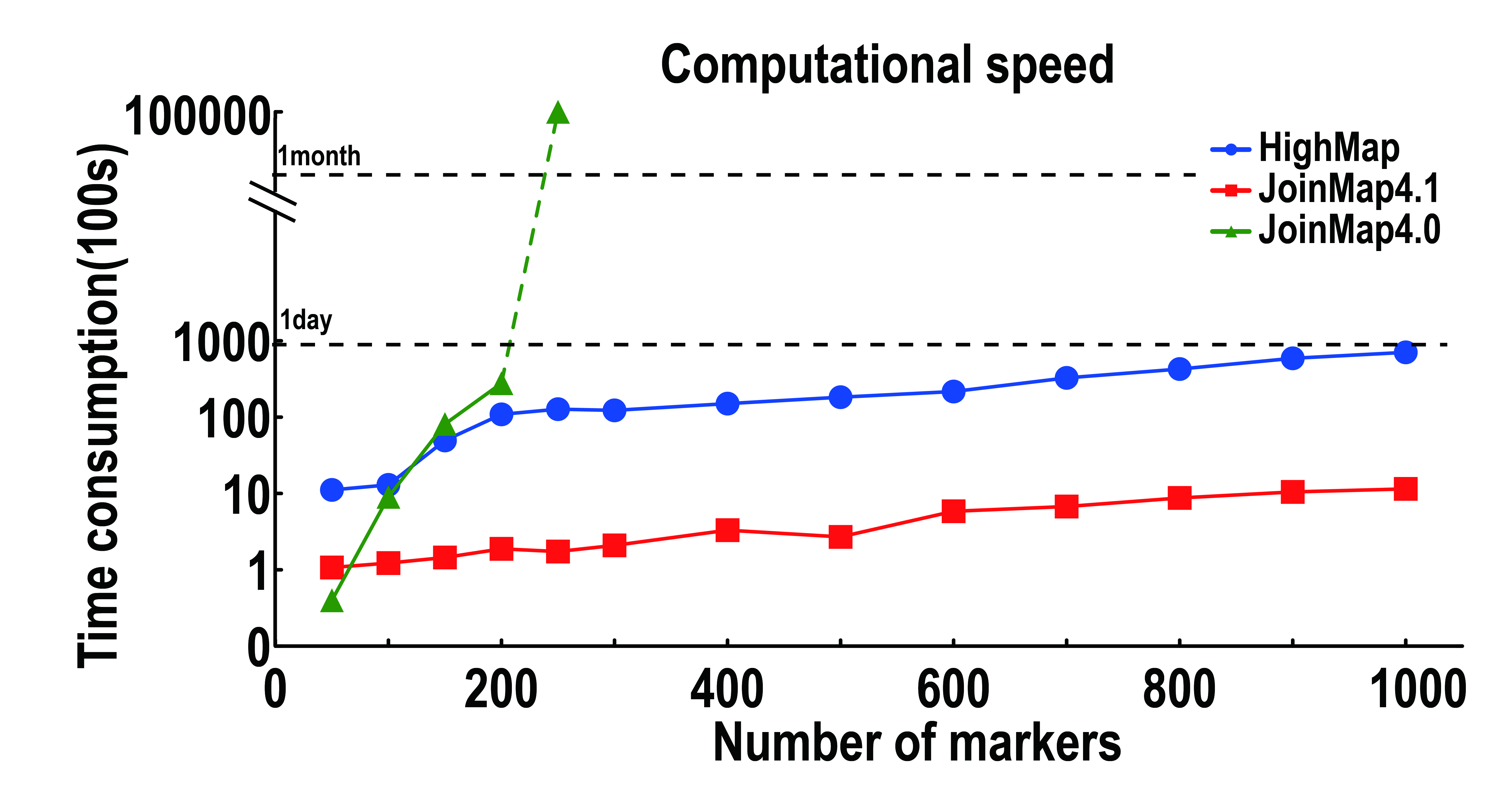

Supplement: Figure S3 — Computational speed of HighMap. Running time was reported as number of 100 seconds. JoinMap4.0 is computationally demanding when marker data contained more than 200 markers. (TIF) [file pone.0098855.s003.tif]

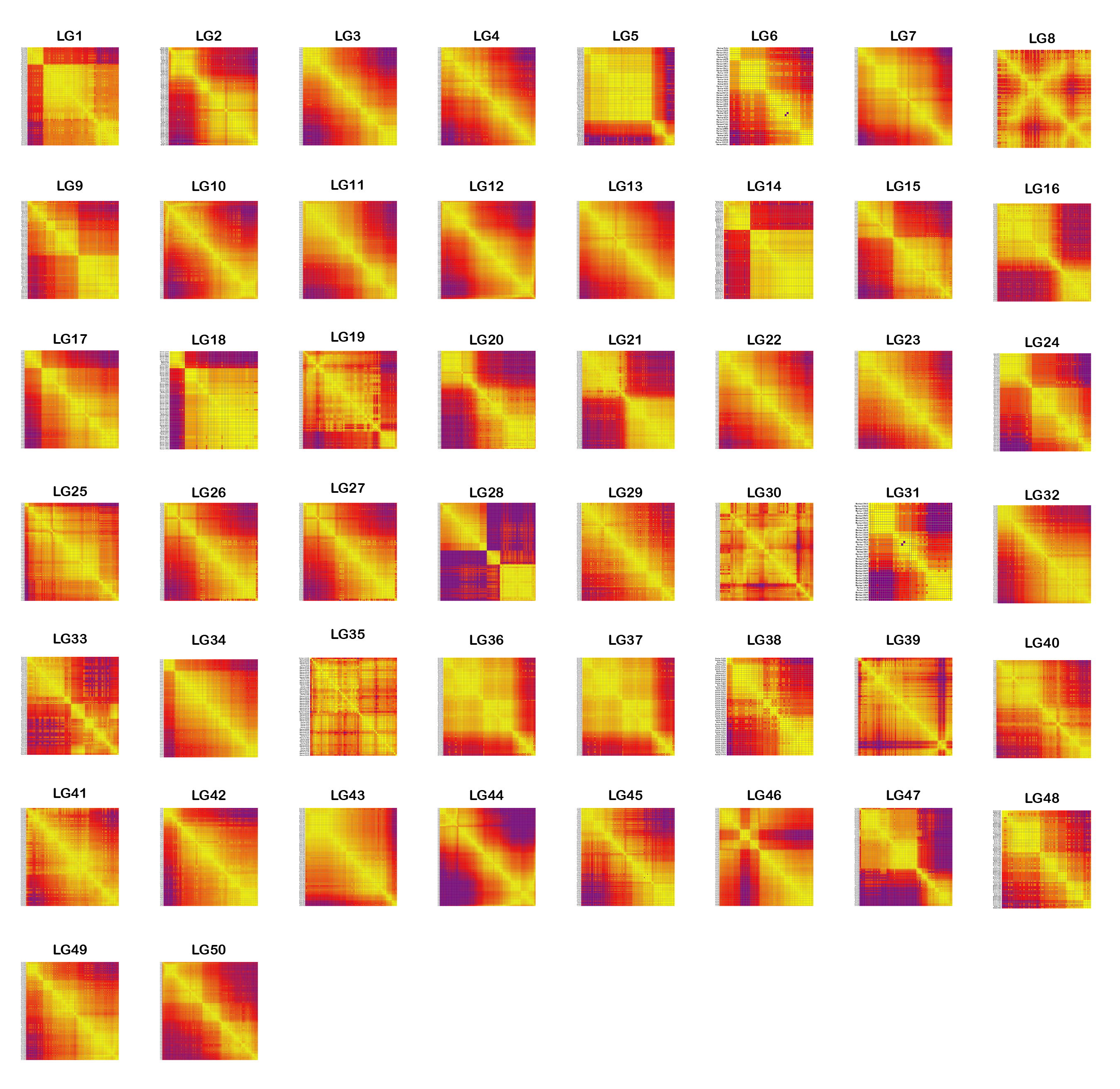

Supplement: Figure S4 — Heat maps of pair-wise recombination of the common carp. Yellow color represents tight linkage; red represents weak linkage; blue represents no linkage. (TIF) [file pone.0098855.s004.tif]

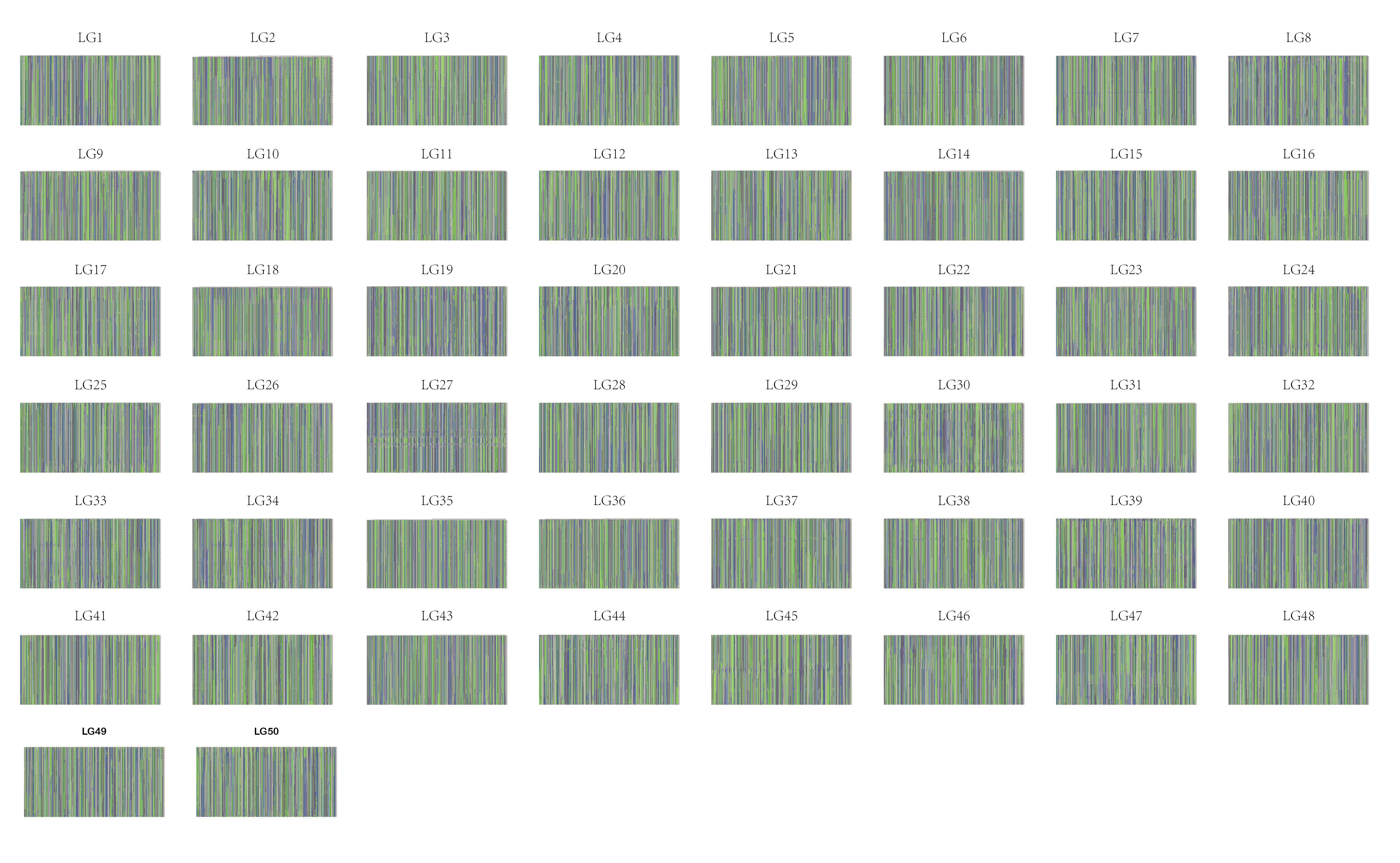

Supplement: Figure S5 — Haplotype maps of the family of common carp consisting of 211 offsprings. Each two columns represent the genotype of an individual. Rows correspond to genetic markers. Green and blue boxes indicate one chromatid from parents; gray boxes indicate missing data. (JPG) [file pone.0098855.s005.jpg]

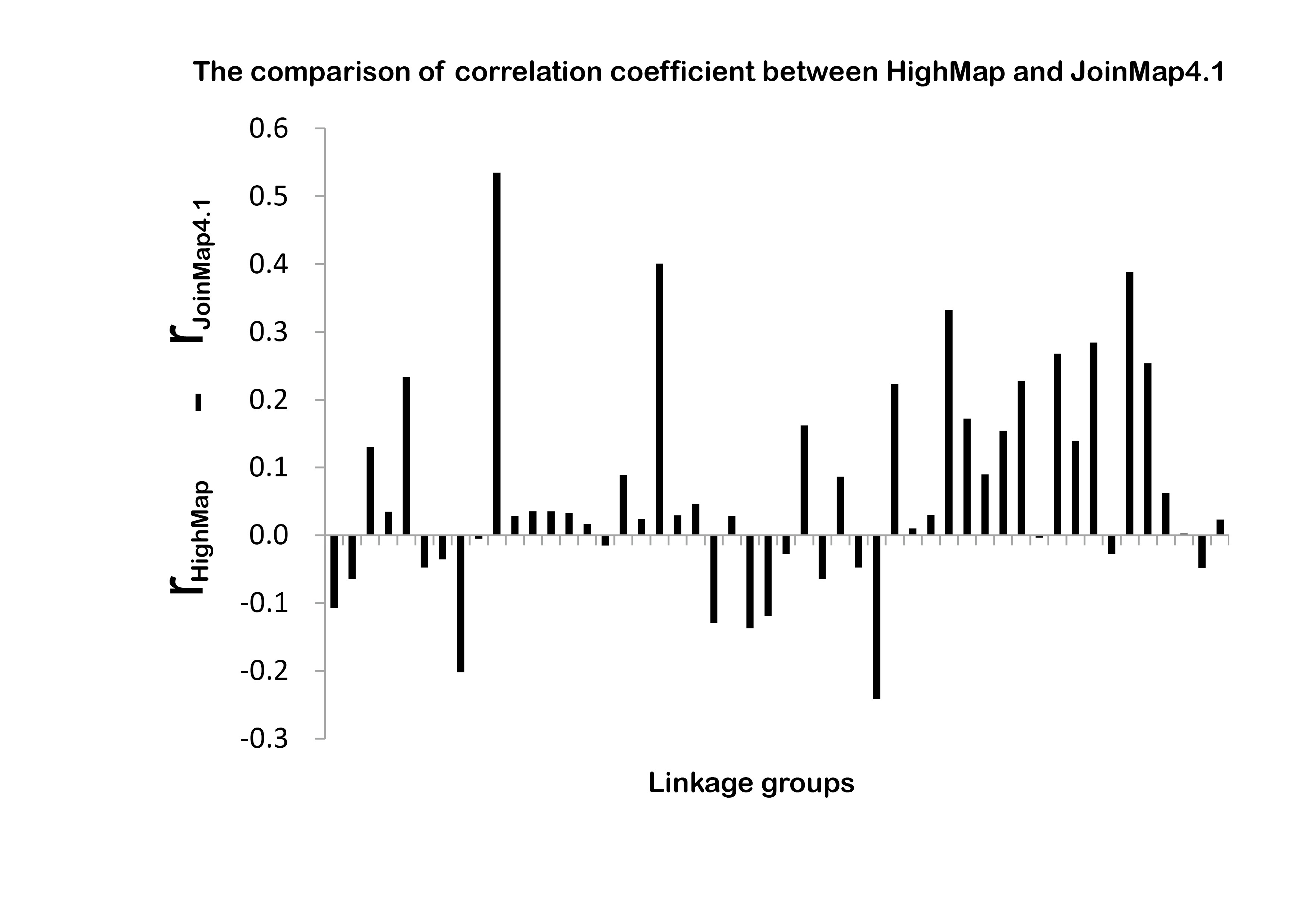

Supplement: Figure S6 — The difference between the correlation coefficient of HighMap and JoinMap4.1. rHighMap indicates the Spearman correlation coefficient between marker order of linkage map estimated by HighMap and genome sequences of zebra fish. rJoinMap4.1 indicates the Spearman correlation coefficient between the marker order of linkage map estimated by JoinMap4.1and the genome sequences of zebra fish. (JPG) [file pone.0098855.s006.jpg]
