# Supplementary material for: Construction and Analysis of High-Density Linkage Map Using High-Throughput Sequencing Data
Source: PLoS One. 2014 Jun 6;9(6):e98855. doi: 10.1371/journal.pone.0098855 (PMC4048240; doi:10.1371/journal.pone.0098855)
Supplement: Table S1 — Segregation patterns of common carp linkage map. (DOC) [file pone.0098855.s007.doc]

**Table S1.** Segregation patterns of common carp linkage map

| Maps | Linkage groups | Segregation patterns | | | | | |
| --- | --- | --- | --- | --- | --- | --- | --- |
|  |  | Total | abxcd | efxeg | lmxll | nnxnp | hkxhk |
| Male | 50 | 5,068 | 123 | 643 | 3,107 | / | 1,195 |
| Female | 50 | 6,897 | 123 | 643 | / | 4,936 | 1,195 |
| Integrated | 50 | 10,004 | 123 | 643 | 3,107 | 4,936 | 1,195 |
