# Supplementary material for: Construction and Analysis of High-Density Linkage Map Using High-Throughput Sequencing Data
Source: PLoS One. 2014 Jun 6;9(6):e98855. doi: 10.1371/journal.pone.0098855 (PMC4048240; doi:10.1371/journal.pone.0098855)
Supplement: Table S2 — Singleton rate of common carp linkage map estimated by HighMap and JoinMap4.1. (DOC) [file pone.0098855.s008.doc]

**Table S2. Singleton rate of common carp linkage map estimated by HighMap and JoinMap4.1**

| Linkage groups | Marker numbers | | |  | Singleton rate (%) | | | | | | |
| --- | --- | --- | --- | --- | --- | --- | --- | --- | --- | --- | --- |
|  |  |  |  |  | HighMap | | |  | JoinMap4.1 | | |
|  | Integrated | Female | Male |  | Integrated | Female | Male |  | Integrated | Female | Male |
| LG1 | 230 | 95 | 174 |  | 0.02 | 0.08 | 0.03 |  | 1.77 | 2.54 | 1.59 |
| LG2 | 88 | 70 | 29 |  | 0.13 | 0.17 | 0.16 |  | 1.82 | 1.9 | 1.7 |
| LG3 | 182 | 161 | 42 |  | 0.05 | 0.11 | 0.02 |  | 1.79 | 1.77 | 2.96 |
| LG4 | 226 | 177 | 88 |  | 0.03 | 0.04 | 0.1 |  | 1.68 | 1.79 | 1.72 |
| LG5 | 153 | 82 | 86 |  | 0.01 | 0.19 | 0.04 |  | 1.37 | 1.5 | 1.58 |
| LG6 | 89 | 44 | 61 |  | 0.06 | 0.04 | 0.1 |  | 1.49 | 1.55 | 1.52 |
| LG7 | 201 | 174 | 39 |  | 0.46 | 0.43 | 0.43 |  | 1.65 | 1.69 | 2.13 |
| LG8 | 208 | 138 | 152 |  | 0.01 | 0.04 | 0.04 |  | 1.7 | 1.79 | 1.6 |
| LG9 | 126 | 90 | 49 |  | 0.05 | 0.06 | 0.09 |  | 2.11 | 2.24 | 1.89 |
| LG10 | 251 | 185 | 118 |  | 0.06 | 0.08 | 0.07 |  | 1.64 | 1.62 | 1.61 |
| LG11 | 176 | 158 | 37 |  | 0.04 | 0.05 | 0.12 |  | 1.44 | 1.46 | 1.92 |
| LG12 | 213 | 181 | 46 |  | 0.06 | 0.08 | 0.06 |  | 1.72 | 1.84 | 1.9 |
| LG13 | 264 | 224 | 65 |  | 0.05 | 0.07 | 0.16 |  | 1.71 | 1.64 | 2.27 |
| LG14 | 106 | 71 | 44 |  | 0.02 | 0.02 | 0.08 |  | 1.22 | 1.42 | 1.46 |
| LG15 | 215 | 169 | 84 |  | 0.04 | 0.04 | 0.14 |  | 2 | 2.08 | 1.83 |
| LG16 | 185 | 149 | 84 |  | 0.15 | 0.1 | 0.23 |  | 1.73 | 1.9 | 1.7 |
| LG17 | 183 | 168 | 30 |  | 0.1 | 0.1 | 1.44 |  | 1.98 | 1.91 | 4.5 |
| LG18 | 111 | 63 | 58 |  | 0.01 | 0.05 | 0.02 |  | 1.53 | 1.66 | 1.36 |
| LG19 | 241 | 160 | 137 |  | 0.1 | 0.15 | 0.08 |  | 2.02 | 2.46 | 1.56 |
| LG20 | 246 | 177 | 128 |  | 0.03 | 0.07 | 0.07 |  | 1.55 | 1.74 | 1.46 |
| LG21 | 156 | 114 | 77 |  | 0.03 | 0.09 | 0.02 |  | 1.7 | 1.69 | 1.69 |
| LG22 | 200 | 173 | 51 |  | 0.08 | 0.07 | 0.17 |  | 1.72 | 1.77 | 2.3 |
| LG23 | 277 | 174 | 177 |  | 0.01 | 0.06 | 0.02 |  | 1.34 | 1.3 | 1.3 |
| LG24 | 143 | 91 | 72 |  | 0.06 | 0.28 | 0.1 |  | 1.6 | 1.66 | 1.82 |
| LG25 | 239 | 165 | 145 |  | 0.11 | 0.26 | 0.18 |  | 1.57 | 1.73 | 1.59 |
| LG26 | 177 | 141 | 72 |  | 0.61 | 0.57 | 0.74 |  | 1.8 | 1.98 | 1.35 |
| LG27 | 230 | 77 | 184 |  | 0.03 | 0.17 | 0.05 |  | 1.4 | 1.79 | 1.49 |
| LG28 | 337 | 214 | 221 |  | 1.67 | 2.31 | 0.76 |  | 1.53 | 1.62 | 1.3 |
| LG29 | 220 | 134 | 142 |  | 0.02 | 0.04 | 0.09 |  | 1.65 | 1.49 | 1.85 |
| LG30 | 154 | 130 | 114 |  | 0.13 | 0.19 | 0.34 |  | 1.53 | 1.57 | 1.37 |
| LG31 | 135 | 41 | 107 |  | 0.02 | 0.1 | 0.01 |  | 1.15 | 1.87 | 0.95 |
| LG32 | 184 | 135 | 84 |  | 0.02 | 0.06 | 0.05 |  | 1.51 | 1.51 | 1.74 |
| LG33 | 183 | 100 | 132 |  | 1.22 | 1.82 | 0.59 |  | 1.46 | 1.66 | 1.44 |
| LG34 | 187 | 166 | 42 |  | 0.05 | 0.05 | 0.24 |  | 1.12 | 1.12 | 1.64 |
| LG35 | 84 | 57 | 41 |  | 0.11 | 0.16 | 0.18 |  | 1.1 | 1.31 | 0.98 |
| LG36 | 129 | 109 | 35 |  | 0.01 | 0.02 | 0.45 |  | 1.33 | 1.42 | 1.22 |
| LG37 | 285 | 201 | 152 |  | 0.01 | 0.03 | 0.07 |  | 1.42 | 1.79 | 1.34 |
| LG38 | 155 | 52 | 121 |  | 0.13 | 0.18 | 0.2 |  | 1.62 | 2.13 | 1.57 |
| LG39 | 251 | 162 | 213 |  | 0.08 | 0.21 | 0.09 |  | 2.08 | 2.07 | 1.71 |
| LG40 | 297 | 183 | 181 |  | 0.01 | 0.06 | 0.05 |  | 1.26 | 1.57 | 1.04 |
| LG41 | 182 | 116 | 119 |  | 0.06 | 0.18 | 0.1 |  | 1.33 | 1.77 | 1.38 |
| LG42 | 223 | 182 | 101 |  | 0.81 | 0.91 | 0.54 |  | 1.87 | 2.05 | 2.2 |
| LG43 | 137 | 99 | 57 |  | 0.01 | 0.03 | 0.44 |  | 1.25 | 1.48 | 1.69 |
| LG44 | 327 | 282 | 68 |  | 0.13 | 0.25 | 0.87 |  | 1.57 | 1.58 | 2.55 |
| LG45 | 230 | 146 | 166 |  | 0.05 | 0.05 | 0.06 |  | 1.52 | 1.52 | 1.5 |
| LG46 | 190 | 155 | 43 |  | 0.05 | 0.07 | 0.31 |  | 1.47 | 1.53 | 2.82 |
| LG47 | 237 | 123 | 156 |  | 0.06 | 0.09 | 0.04 |  | 1.61 | 1.77 | 1.56 |
| LG48 | 224 | 70 | 180 |  | 0.41 | 0.35 | 0.42 |  | 1.26 | 1.31 | 1.33 |
| LG49 | 210 | 122 | 123 |  | 0.02 | 0.07 | 0.07 |  | 1.66 | 1.79 | 1.58 |
| LG50 | 327 | 247 | 141 |  | 0.86 | 0.84 | 1.39 |  | 1.9 | 1.77 | 2.42 |
