# Supplementary material for: Construction and Analysis of High-Density Linkage Map Using High-Throughput Sequencing Data
Source: PLoS One. 2014 Jun 6;9(6):e98855. doi: 10.1371/journal.pone.0098855 (PMC4048240; doi:10.1371/journal.pone.0098855)
Supplement: Table S3 — Genetic distance of common carp linkage map estimated by HighMap and JoinMap4.1. (DOC) [file pone.0098855.s009.doc]

**Table S3. Genetic distance of common carp linkage map estimated by HighMap and JoinMap4.1**

| Linkage groups | Marker numbers | | |  | Distances (cM) | | | | | | |
| --- | --- | --- | --- | --- | --- | --- | --- | --- | --- | --- | --- |
|  |  |  |  |  | HighMap | | |  | JoinMap4.1 | | |
|  | Integrated | Female | Male |  | Integrated | Female | Male |  | Integrated | Female | Male |
| LG1 | 230 | 95 | 174 |  | 96.2 | 101 | 91.3 |  | 767.6 | 700.7 | 770.2 |
| LG2 | 88 | 70 | 29 |  | 83.9 | 125.1 | 21.7 |  | 327.6 | 433.8 | 114 |
| LG3 | 182 | 161 | 42 |  | 72.8 | 102 | 42.8 |  | 699.8 | 731.8 | 491.6 |
| LG4 | 226 | 177 | 88 |  | 93.9 | 131.9 | 52 |  | 667.9 | 910.7 | 301.1 |
| LG5 | 153 | 82 | 86 |  | 84.3 | 77.3 | 67.9 |  | 449.1 | 504.2 | 343.5 |
| LG6 | 89 | 44 | 61 |  | 52.5 | 61.2 | 43.3 |  | 249.2 | 203 | 230.3 |
| LG7 | 201 | 174 | 39 |  | 70.3 | 114 | 15.4 |  | 490.1 | 840.5 | 136.5 |
| LG8 | 208 | 138 | 152 |  | 153.3 | 172.3 | 133.3 |  | 659.9 | 660.7 | 596.7 |
| LG9 | 126 | 90 | 49 |  | 80.7 | 101 | 52.4 |  | 464.5 | 554.6 | 319.1 |
| LG10 | 251 | 185 | 118 |  | 112.9 | 132.3 | 71.9 |  | 717.7 | 908.1 | 499.8 |
| LG11 | 176 | 158 | 37 |  | 74.7 | 93.1 | 44.8 |  | 478.7 | 636 | 176.2 |
| LG12 | 213 | 181 | 46 |  | 135.2 | 169.6 | 76.9 |  | 756.4 | 984.8 | 248.8 |
| LG13 | 264 | 224 | 65 |  | 105.7 | 132.4 | 74.2 |  | 917.6 | 1,025.30 | 541.7 |
| LG14 | 106 | 71 | 44 |  | 64.9 | 71 | 37.1 |  | 285.9 | 283.8 | 221.6 |
| LG15 | 215 | 169 | 84 |  | 115.9 | 144.2 | 77.4 |  | 779.8 | 1,015.10 | 389.9 |
| LG16 | 185 | 149 | 84 |  | 115.5 | 134.3 | 90.4 |  | 660.7 | 760.3 | 460.3 |
| LG17 | 183 | 168 | 30 |  | 129.1 | 142.1 | 102.1 |  | 619.1 | 894.8 | 324 |
| LG18 | 111 | 63 | 58 |  | 72.5 | 70.4 | 67.5 |  | 289.9 | 293.1 | 233.4 |
| LG19 | 241 | 160 | 137 |  | 231 | 321 | 77 |  | 924.5 | 1,278.60 | 510 |
| LG20 | 246 | 177 | 128 |  | 144.8 | 161.4 | 104.2 |  | 744.8 | 948.7 | 485.8 |
| LG21 | 156 | 114 | 77 |  | 85.7 | 99.9 | 63.1 |  | 497.3 | 530.5 | 370.8 |
| LG22 | 200 | 173 | 51 |  | 96.7 | 134.1 | 41.5 |  | 631.3 | 807.4 | 257 |
| LG23 | 277 | 174 | 177 |  | 97 | 100.8 | 93.2 |  | 659.5 | 679.1 | 578.2 |
| LG24 | 143 | 91 | 72 |  | 100.2 | 80.4 | 97.3 |  | 430.5 | 431 | 353.6 |
| LG25 | 239 | 165 | 145 |  | 159.2 | 180.9 | 136.5 |  | 685.7 | 745.1 | 565.8 |
| LG26 | 177 | 141 | 72 |  | 90.7 | 135.3 | 35.2 |  | 566 | 785.9 | 196.4 |
| LG27 | 230 | 77 | 184 |  | 106.4 | 84 | 128.7 |  | 741.4 | 401.1 | 909 |
| LG28 | 337 | 214 | 221 |  | 373.5 | 641.2 | 91.1 |  | 968.7 | 1,045.70 | 764.7 |
| LG29 | 220 | 134 | 142 |  | 100.3 | 78.1 | 121.3 |  | 703.7 | 490.5 | 731.7 |
| LG30 | 154 | 130 | 114 |  | 212.4 | 274.8 | 148.9 |  | 605.8 | 689.3 | 417.2 |
| LG31 | 135 | 41 | 107 |  | 72.6 | 77.9 | 63.4 |  | 5,329.50 | 10,297.30 | 273.2 |
| LG32 | 184 | 135 | 84 |  | 80.3 | 101.5 | 54 |  | 585.2 | 606.2 | 350.1 |
| LG33 | 183 | 100 | 132 |  | 120.5 | 167 | 66.7 |  | 574.5 | 552.4 | 495.1 |
| LG34 | 187 | 166 | 42 |  | 76.6 | 120.3 | 28.1 |  | 327.4 | 482.2 | 106.1 |
| LG35 | 84 | 57 | 41 |  | 172.9 | 185.2 | 81.4 |  | 164.5 | 187.1 | 93.2 |
| LG36 | 129 | 109 | 35 |  | 84.8 | 100.3 | 43.2 |  | 391.6 | 474.9 | 168.6 |
| LG37 | 285 | 201 | 152 |  | 119.3 | 153.6 | 84 |  | 740 | 953 | 453.1 |
| LG38 | 155 | 52 | 121 |  | 117.1 | 84.1 | 123 |  | 446.8 | 323.9 | 513.8 |
| LG39 | 251 | 162 | 213 |  | 215.3 | 314.7 | 110.3 |  | 1,067.80 | 1,079.60 | 862.3 |
| LG40 | 297 | 183 | 181 |  | 116.7 | 140.7 | 84.8 |  | 765.8 | 946.7 | 560.7 |
| LG41 | 182 | 116 | 119 |  | 85.9 | 135 | 26.7 |  | 381.2 | 513 | 210.2 |
| LG42 | 223 | 182 | 101 |  | 88 | 118 | 55.1 |  | 779.9 | 957.5 | 484.4 |
| LG43 | 137 | 99 | 57 |  | 59.7 | 67.8 | 51 |  | 366.4 | 418.1 | 303.8 |
| LG44 | 327 | 282 | 68 |  | 175.9 | 211.7 | 140.2 |  | 932.1 | 1,274.70 | 527.7 |
| LG45 | 230 | 146 | 166 |  | 156.2 | 118.2 | 173.4 |  | 10,742.00 | 677.3 | 10,731.20 |
| LG46 | 190 | 155 | 43 |  | 118.9 | 138.6 | 74.9 |  | 10,553.70 | 10,648.70 | 458.7 |
| LG47 | 237 | 123 | 156 |  | 153.3 | 175.2 | 110.7 |  | 709.9 | 665 | 639.1 |
| LG48 | 224 | 70 | 180 |  | 98.1 | 73.1 | 107.6 |  | 548.4 | 364.4 | 680 |
| LG49 | 210 | 122 | 123 |  | 101.1 | 107.7 | 93.1 |  | 629 | 638 | 583.9 |
| LG50 | 327 | 247 | 141 |  | 182.6 | 190.4 | 174.8 |  | 1,073.50 | 1,184.60 | 915.3 |
